# Cloning and promoter analysis of palladin 90-kDa, 140-kDa, and 200-kDa isoforms involved in skeletal muscle cell maturation

**DOI:** 10.1186/s13104-020-05152-9

**Published:** 2020-07-03

**Authors:** Boimpoundi Eunice Flavie Ouali, Tzu-Yu Liu, Chun-Yen Lu, Pei-Yuan Cheng, Chao-Li Huang, Chun-Chun Li, Yu-Chung Chiang, Hao-Ven Wang

**Affiliations:** 1 Department of Life Sciences, College of Biosciences and Biotechnology, National Cheng Kung University, No. 1 University Road, East Dist.,, Tainan City, 701 Taiwan (R.O.C.); 2 Institute of Tropical Plant Sciences, College of Biosciences and Biotechnology, National Cheng Kung University, Tainan, Taiwan (R.O.C.); 3 Department of Biological Sciences, National Sun Yat-sen University, 70 Lienhai Rd., Kaohsiung, 80424 Taiwan (R.O.C.); 4 Department of Biomedical Science and Environment Biology, Kaohsiung Medical University, Kaohsiung, Taiwan (R.O.C.); 5 Center for Bioscience and Biotechnology, National Cheng Kung University, Tainan, Taiwan (R.O.C.); 6 Marine Biology and Cetacean Research Center, National Cheng Kung University, Tainan, Taiwan (R.O.C.)

**Keywords:** Palladin, Cytoskeleton, Promoter analysis, RNA isoform

## Abstract

**Objective:**

Palladin is a ubiquitous phosphoprotein expressed in vertebrate cells that works as a scaffolding protein. Several isoforms deriving from alternative splicing are originated from the palladin gene and involved in mesenchymal and muscle cells formation, maturation, migration, and contraction. Recent studies have linked palladin to the invasive spread of cancer and myogenesis. However, since its discovery, the promoter region of the palladin gene has never been studied. The objective of this study was to predict, identify, and measure the activity of the promoter regions of palladin gene.

**Results:**

By using promoter prediction programs, we successfully identified the transcription start sites for the *Palld* isoforms and revealed the presence of a variety of transcriptional regulatory elements including TATA box, GATA, MyoD, myogenin, MEF, Nkx2-5, and Tcf3 upstream promoter regions. The transcriptome profiling approach confirmed the active role of predicted transcription factors in the mouse genome. This study complements the missing piece in the characterization of palladin gene and certainly contributes to understanding the complexity and enrollment of palladin regulatory factors in gene transcription.

## Introduction

Palladin is a cytoskeletal associated protein that plays a fundamental role in human and animal cell morphology and adhesion. It was simultaneously and independently identified and characterized by two groups of researchers in early 2000 [1, 2]. At that time, the protein is known to organize and stabilize the actin cytoskeleton and focal adhesions at the cell–cell and cell–matrix junctions in embryonic and fibroblast cells and tissues [2]. Subsequently, the mouse palladin gene (*Palld*) was described as a complex structure that spans approximately 400 kb and is composed of 25 exons coding for several isoforms [3, 4] that highly interact with various cytoskeletal associated proteins [1, 5–8]. The contribution of palladin to various biological, physiological processes and signaling pathways within cells is not undeniable [9–11] and has been partially elucidated in our laboratory.

From online databases, we found that the human palladin gene generates about 14 isoforms against four for the mouse palladin gene. Investigations are still carried out to classify proteins that regulate or directly interact with palladin with a substantial impact on cell structure and function. However, no study has ever identified the activity of promoter regions for the three well-characterized palladin isoforms (90-kDa, 140-kDa, and 200-kDa). Here, we used bioinformatics tools to predict the promoter region of *Palld* and describe potential palladin regulatory factors.

## Main text

### Methods

#### Cell culture

C2C12 cells, a mouse myogenic cell line, were purchased from the American Type Culture Collection (ATCC^®^ CRL-1772™) and maintained in complete growth medium (GM) containing Dulbecco’s modified Eagle’s medium (DMEM, Gibco) with 10% fetal bovine serum (Gibco), and 1% penicillin/streptomycin (Gibco) in a humidified atmosphere of 5% CO_2_ at 37 °C. Differentiation of myoblasts into myotubes was induced by exchanging GM to differentiation medium (DM) containing DMEM and 2% horse serum (Gibco). Fresh DM was replaced every day after differentiation induction from Day 0 (D0) to Day 5 (D5).

#### Promoter analysis of mouse gene *Palld*

DNA sequences of *Palld* (Gene ID: 72333) were obtained from the National Center for Biotechnology Information (NCBI) and Mouse Genome Informatics (MGI) databases. Identification of the Transcription Start Site (TSS) of the three palladin isoforms 90-kDa, 140-kDa, and 200-kDa was performed using the Ensembl genome browser. DNA sequences of approximately − 3 kb from the start codon of each isoform were analyzed by using three online promoter prediction programs including Promoter 2.0 Prediction Server, Neural Network Promoter Prediction, and PROMOTER SCAN. To compare these gene sequences with multiple vertebrate genomes, an Evolutionary Conserved Regions (ECR) Browser database was used. Prediction of Transcription Factor Binding Sites in DNA sequences was performed by using MATCH and PROMO tools.

#### DNA construction of luciferase reporter and DNA transfection

Multiple DNA fragments of predicted *Palld* promoter regions were amplified from mouse genomic DNA (C57BL/6 J) and subcloned into the pGL4.17 [luc2/neo] vector (Promega, USA) with a downstream tagged luciferase gene. DNA fragments of palladin isoform 90-kDa, 140-kDa, and 200-kDa were respectively inserted into SacI and BglII, KpnI and EcoRV, and KpnI and HindIII sites of the multiple cloning region of the vector.

C2C12 cells were transfected with various palladin promoter constructs tagged with a luciferase gene or pGL4.74 [hRluc, TK] empty vector (Promega, USA) as a control plasmid by using Lipofectamine 2000 reagent (Invitrogen) and further incubated for 4 days following differentiation induction.

#### Luciferase reporter assay

The Dual-Glo Luciferase Reporter Assay System Kit (Promega) was used according to the manufacturer’s protocol. Luminescence of each sample was quantified on a Tecan Infinite F200 Pro ELISA reader (USA). Values were normalized to the control vector, and the results were expressed as the ratio of firefly luciferase activity to *Renilla* luciferase activity for each construct.

#### RNA isolation and sequencing

Total RNA from C2C12 cells was extracted by using High Pure RNA Isolation Kit (Roche) according to the manufacturer’s instructions. The RNA concentration was determined using NanoDrop 2000 spectrophotometer (Thermo fisher) and RNA purity and integrity was evaluated using Experion™ Automated Electrophoresis System. The samples were submitted to Yourgene Bioscience (Taiwan) for sequencing. The strand specific Poly-A mRNA libraries were generated and sequenced by using Illumina HiSeq™ 4000 (NovogeneAIT, Singapore) platform and procedure. Quality trimming was performed and paired-end reads were mapped to the mouse reference genome GRCm38.p6 using TopHat software. Assembled transcripts were acquired by using Cufflinks and transcriptome was assembled via Cuffmerge. Gene expression levels were normalized by computing the Fragments Per Kilobase of transcript per Million mapped reads (FPKM). Because of the downstream usage of sequencing results not discussed in the present work, the data have not yet been made available in a public repository. For sequencing data partially used to support our analysis, raw data can be accessed on request.

#### Quantitative PCR

The same RNA pools used for RNA seq were employed for qPCR. Complementary DNA (cDNA) was synthesized using iScript cDNA Synthesis Kit (Bio-Rad). The primers used were listed in Additional file 1. Each reaction tubes contained Power SYBR Green PCR Master Mix (Applied Biosystems), Forward and Reverse primer at the final concentration of 500 nM, 1 µl of synthetized cDNA, and PCR grade water to a total volume of 10 µl. Each sample was tested in duplicate using the StepOnePlus Real-Time PCR System (Applied Biosystems) at the following conditions: 95 °C for 10 min, 40 cycles of 95 °C for 15 s, and 60 °C for 1 min. Amplification specificity was assessed by melting curve. The Ct values obtained were normalized against the Adaptor Related Protein Complex 3 Subunit Delta 1 (Ap3d1) endogenous control and expression of mRNA calculated using the 2^−∆Ct^, a derivative of Livak method [12].

#### Statistical analysis

Two-way repeated-measures analysis of variance (ANOVA) with Bonferroni corrections for multiple group comparisons at various time point were calculated. Values of *p* ≤ 0.05 were considered statistically significant.

### Results

#### Identification of predictive *Palld* promoter regions

To predict and identify the nature of regulatory elements controlling the activation or repression of palladin isoforms, gene fragments of 3000 bp upstream the first exon were generated and the palladin gene promoter region analyzed. The promoter regions were predicted to be within the − 2917 bp/+ 83 bp, − 2721 bp/+ 279 bp, and − 2871 bp/+ 129 bp, respectively for palladin isoform 90-kDa, 140-kDa, and 200-kDa. Promoter 2.0 Prediction Server data indicate the presence of at least one putative eukaryotic polymerase II binding site within each isoform (Additional file 2: Appendix A). Through the Neural Network Promoter Prediction program, various polymerase II binding sites were observed at different positions along with TATA box sequences (Additional file 2: Appendix B). While the existence of TATA box-binding sites implies that of a polymerase II binding site, it did not necessarily refer to the exact transcription initiation location. Web Promoter Scan program revealed potential transcription factor binding sites for the 90-kDa isoform but failed for the prediction of 140-kDa and 200-kDa (Additional file 2: Appendix C).

**Fig. 1:**
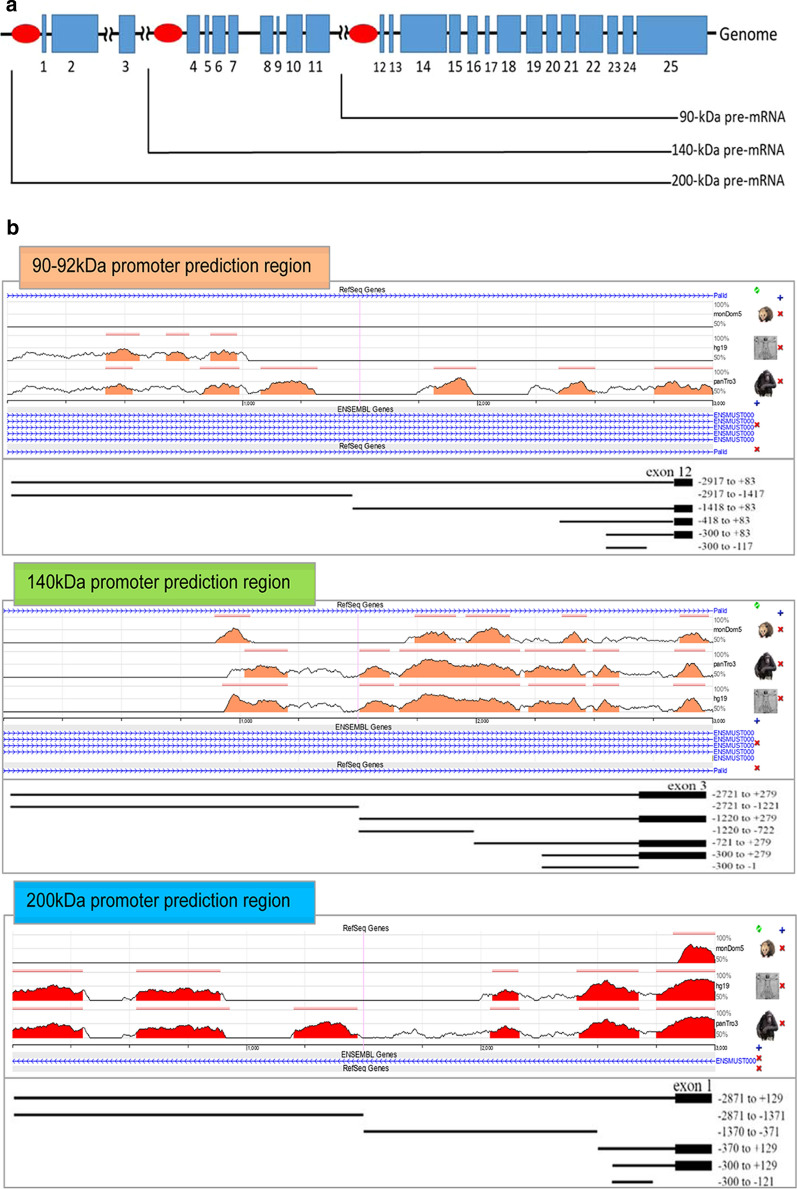
Promotor prediction region of palladin isoforms. **a** Schematic presentation of the murine palladin gene structure. Blue closed boxes show translated regions of different splice variants and red elliptical shapes show predictive localization of each splice variant promoter region. **b** visualization of Conserved Regions (ECRs) within the predicted promoter region of palladin isoforms of different species. Mouse palladin reference gene was compared to palladin genes from Opossum, Human, and Chimpanzee

To further identify conserved elements in *Palld* between mouse and other animal species, DNA sequences were aligned and submitted to ECR browser. A schematic representation of the mouse palladin gene indicated the location of isoform transcripts and the promoter region of each transcript (Fig. 1a). Several conserved regions between mouse and human were identified. There were no similarities between mouse (*Mus musculus*) and opossum (*Monodelphis domestica*) for palladin 90-kDa isoform however the mouse shared three conserved regions with the human (*Homo sapiens*) and six intronic regions with the chimpanzee (*Pan troglodytes*) (Fig. 1b). Six intronic regions of 140-kDa isoform were conserved between mouse, human, and chimpanzee. The 200-kDa isoform presented five common conserved regions with a unique region shared between mouse and opossum.

**Fig. 2:**
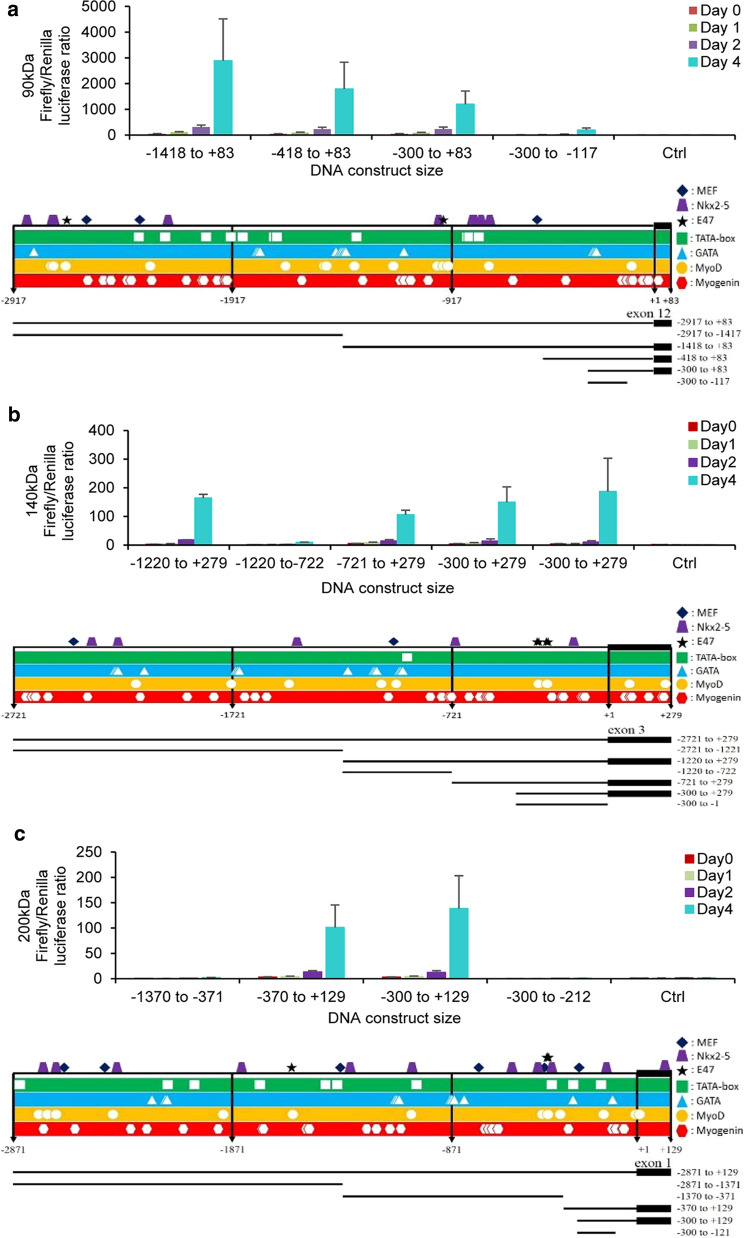
Palld gene predictive promoter regions location. Luciferase reporter activities at distinct differentiation days (D0, D1, D2, D4) of various Palld gene constructs comprising potential promoter region and representative gene regulatory sequences of palladin. **a** 90-kDa isoform, **b** 140-kDa isoform and **c** 200-kDa isoform. Luciferase activity measured in wells without cells was used as control (Ctrl). Gene fragments were designed with positive and negative numbers respectively representing number of bp upstream and downstream the first coding region of each isoform. The variation of Firefly/Renilla luciferase ratio between constructs were not statistically different considering the mean of three repeats

By using MATCH and PROMO software, the promoter regions were restricted up to 1.5 kb for each isoform. For the targeted constructs, substantial high levels of luciferase activity were observed 4 days after C2C12 cell transfection. Promoter regions were delineated based on promoter activity increase in comparison to the activity of the promoter-free construct. Relative firefly/*Renilla* luciferase activity was measured for the palladin 90-kDa isoform (Fig. 2a), palladin 140-kDa isoform (Fig. 2b), and palladin 200-kDa isoform (Fig. 2c) with their respective gene representations and landmarks. These outcomes indicate multiple potential TSS for palladin isoforms with sequences located at the region between − 1418 and + 83 bp of exon 12 for the 90-kDa isoform, -300 and − 1 bp upstream exon 3 for the 140-kDa isoform and − 300 to + 129 bp of exon 1 for the 200-kDa isoform.

#### RNA-Seq transcriptome analysis

**Fig. 3:**
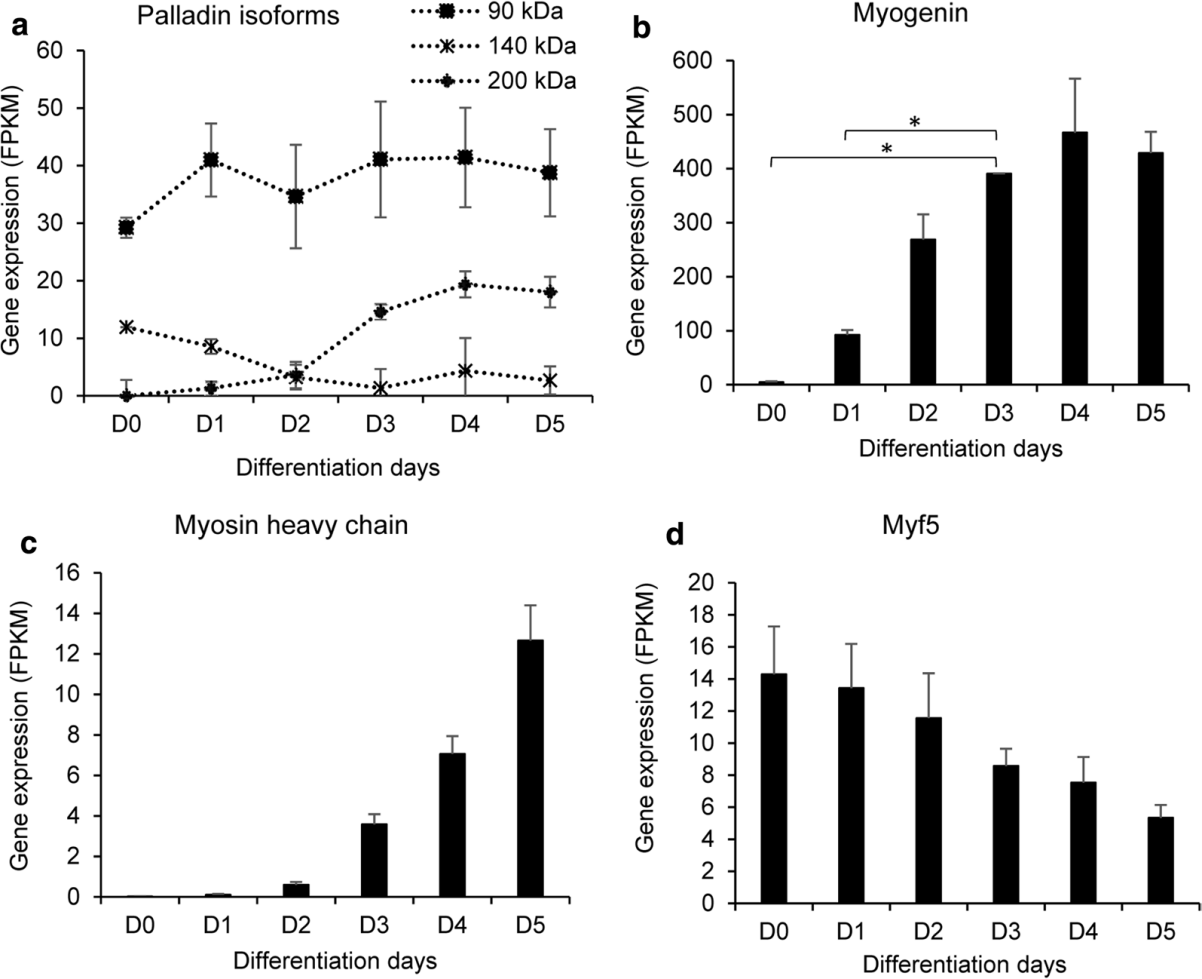
Palladin and myogenic genes mRNA modulation during C2C12 differentiation. Six samples generated from C2C12 differentiated from D0 to D5 were submitted for RNA-Seq analysis and results presented as Fragments Per Kilobase of transcript per Million mapped reads (FPKM) ± SEM, n = 3 independent biological replicates. **a** Palladin isoforms, **b** myogenin, **c** Myosin heavy chain were significantly upregulated and **d** Myf5 was downregulated during cell differentiation. (*) *p* value < 0.05

To investigate mRNA dynamics inherent to myoblast cell ongoing differentiation for the specific palladin isoforms and some myogenic genes, C2C12 RNA transcripts were analyzed. The expression of palladin isoforms was determined according to the base pair count which differentiate multiple predicted and known palladin isoforms. The expression of genes involved in cell maturation differentially and significantly changed during myogenesis. The highest expressing transcript was palladin 90-kDa mRNA compared to the other isoforms (Fig. 3a). The expression of 90-kDa and 200-kDa isoforms increased during cell differentiation, which was opposite to that of the 140-kDa isoform. Genes encoding muscle hypertrophic and sarcomeric proteins, for example myogenin (Fig. 3b) and myosin heavy chain (Fig. 3c) were upregulated while myf5 (Fig. 3d) was downregulated.

Additionally, RNA transcripts analysis confirmed the presence and the activity of predicted regulatory elements such as Myocyte enhancer factor–2 (MEF2)-Mef2a, Mef2b, Mef2c, homeobox protein Nkx2-5, and Tcf3 in an inclusive way (Additional file 3). The qPCR validated and correlated with transcriptome findings in terms of mRNA expression trend (Additional file 4).

### Discussion

Here, we address the gap of knowledge about the promoter sites driving the expression of three major forms of palladin isoforms. Analysis using Promoter 2.0 Prediction Server, Neural Network Promoter Prediction, and PROMOTER SCAN software disclosed different outcomes that might be caused by different computational algorithms used by each promoter prediction software which primarily relies on specific motifs included in the core promoter [13–15].

MATCH and PROMO software elicited multiple binding sites of seven transcription factors including a; TATA box (core sequence 5′-TATAAA-3′), GATA, MyoD, myogenin, MEF, Nkx2-5 and E47 sites. Most of the predicted transcription factors are involved in the maturation process of myoblast cells into myofibers. As reported previously, C2C12 cells myoblast remodeling during differentiation requires activation of myogenic regulatory factors (MRF) for example myogenic termination gene (MYOD), myogenin, MRF4, and myogenic factor 5 (MYF5) [16–18]. In studies of the molecular mechanism underlying heart development, Nkx2.5, MEF family, GATA 4, and GATA 6 were found to be the key regulatory elements in cardiac muscle [19–21]. MEF2 site is essential for the amplification of *Mef2c* involved in skeletal muscle development [22]. Additionally, analysis of the promoters of the actin-related group of serum response factor (SRF) with scaffolding or contractile functions has indicated that approximately 43% of the involved genes in the mouse contains TATA box sequences within the core promoter region [23]. In a cascade regulation process induced by the cytokine transforming growth factor beta (TGF-β), E2A immunoglobulin enhancer‐binding factors E12 and E47 regulate palladin cytoskeletal protein [24]. These findings confirmed our results regarding the identification of active transcription regulation elements on targeted *Palld* gene sequences. Luciferase assay showed various activities of the putative promoter core region for the three isoforms which were highly triggered during differentiation. Yet, discrepancies between palladin 140-kDa mRNA measured and the transcript level in luciferase activity were assumed to be normal since luciferase activity could not evaluate protein absolute concentration. *Palld* has indeed different promoter regions that drive the expression of each isoform and that carry TSS sites and transcriptional regulator factors. The downstream activity of *Palld* ultimately modulates cells’ differentiation or myogenesis within skeletal muscle cells supporting mammalian embryogenesis and development. The ability to control the expression of the palladin gene could then regulate biological events in which the protein is involved.

## Limitations

Transcriptome analysis of C2C12 cells’ ongoing differentiation revealed the functional role of elements predicted to regulate palladin isoform gene. However, at this point in the investigation, we are unable to experimentally attest the presence of the predictive regulatory elements within the promoter regions of the *Palld* gene. Thus, these results might be considered as a starting point for a more explorative study.

## Supplementary information

**Additional file 1: Table S1.** Oligonucleotides used for qPCR.

**Additional file 2: Table S2.** Transcription start sites (TSS) prediction by Promoter 2.0 Prediction Server.

**Additional file 3: Figure S1.** Transcription factors general expression.

**Additional file 4: Figure S2.** Expression of palladin isoforms and myogenic genes.

## Data Availability

All data generated or analyzed during this study are included in this published article and its supplementary information files.

## Abbreviations

DMEM: Dulbecco’s modified Eagle’s medium
GM: Growth medium
DM: Differentiation medium
mRNA: Messenger Ribonucleic acid
TSS: Transcription start site
FPKM: Fragments Per Kilobase of transcript per Million mapped reads
Ap3d1: Adaptor Related Protein Complex 3 Subunit Delta 1
MEF2: Myocyte enhancer factor-2
MRF: Myogenic regulatory factors
MYOD: Myogenic termination gene
Myf5: Myogenic factor 5
