# Supplementary material for: Cloning and promoter analysis of palladin 90-kDa, 140-kDa, and 200-kDa isoforms involved in skeletal muscle cell maturation
Source: BMC Res Notes. 2020 Jul 3;13:321. doi: 10.1186/s13104-020-05152-9 (PMC7333403; doi:10.1186/s13104-020-05152-9)
Supplement: Supplementary file 1 — Additional file 1: Table S1. Oligonucleotides used for qPCR. [file 13104_2020_5152_MOESM1_ESM.pdf]

Additional file 1.
Table S1. Oligonucleotides used for qPCR

Gene	Forward	Reverse
90-kDa	CAGATGGGACTTTTCCGCTC	ACTTGGTTCTGCAGCTGCTG
140-kDa	TGCTGCCTGTGCATTTTCCC	AGCTTTCGCTGTCAGAGTCC
200-kDa	CATCCAGAAACTGAGGAGCC	AGCTTTCGCTGTCAGAGTCC
Myogenin	GGGCAATGCACTGGAGTTCG	GTGCAGATTGTGGGCGTCTG
MyHC 2a-myosin	CAAGAGACAAGCTGAGGAGGC	CATCGGGACAGCCTTACTCTT
Myf5	TCCAACTGCTCTGACGGCAT	GCAATCCAAGCTGGACACGG
Ap3d1	GCATCCGCAACCACAAGGAG	AGCCCAGCTGATGTCGTACC
