# Supplementary material for: Cloning and promoter analysis of palladin 90-kDa, 140-kDa, and 200-kDa isoforms involved in skeletal muscle cell maturation
Source: BMC Res Notes. 2020 Jul 3;13:321. doi: 10.1186/s13104-020-05152-9 (PMC7333403; doi:10.1186/s13104-020-05152-9)
Supplement: Supplementary file 2 — Additional file 2: Table S2. Transcription start sites (TSS) prediction by Promoter 2.0 Prediction Server. [file 13104_2020_5152_MOESM2_ESM.pdf]

Additional file 2

Table S2. Transcription start sites (TSS) prediction by Promoter 2.0 Prediction Server

Isoform	Transcription start site	Sequence (5'→ 3')
90-92-kDa	1600	TTTTAGAGAAATTGCCAGGGGCCCAGCCAGACCTCTTAGTTGGACTCCAG
140-kDa	500	ACAGGATCTCTATGTAGTCCTGATGGTCCTAGAACTCACATGTTAAACAG
	1300	AAGGGGCTTCTAACCACCCCTTCTGGAGAGGAGGAAGAAGCCAGTGAAAC
	1800	ATAACTTTATTAGTCCCTCATGAGTTTCTTCTTCAAATTTCTAAATCCAA
	2500	AACTTGCTTGTGGCTGGGACTACCTTGTCACTTCAGTGGTGTGTAATTGT
200-kDa	600	GTGAGAGGAAGATGTCTAAAGATGGGCAATGTTTGAAGGATTTGTTCTGA
	1000	AATAGTGAAGTTGTCAATTAAGACTATGTACAAGGCAAGACCTGGAGGCC
	2800	GTAGAGTAAGAGAACAGATGAGTGTCTAAGGCCACAGTTGTCTCACTACC

Appendix B. Transcription start sites (TSS) prediction by Neural Network Promoter Prediction program

Isoform	Transcription start site	Sequence (5'→ 3')
90-92-kDa	964-1014	GTTAGGCTTTAAAAAATAGGCCACAGAATCGTATGTAAACAATGACCTAA
	1011-1061	TAATCTATGTTTTAAATATGTGCTTAAACACACACACACAAAAGTCTGGA
	1483-1533	ATCAGAGCATTTAAATGAGTGGTCTAATTGGTACTGACTGATACTTCTTA
	1710-1760	ATGCTAGCAGGAAAAATGGGGCAGAAATAGCTGTGATCAGAGTGCTGGCC
	2352-2402	TGTGCCTTTTTATTTAAAGGCTGCACTTTCTGAGCCTGCCAGTCTGCCCC
	2712-2762	CGTGGCAAGGTTAAAAGGGGGCGGACACCCTAACCTGGGGCTGCTCCCCA
	2880-2930	GATGCTTGGCGTATAAAGCGGGGGTACCTGCCCGGCGCAGCCGGAGGAGCC
140-kDa	230-280	TCTCTTCCTAAGTATAAATGCCACGGTGGGAGGAGGAGCCAATTTCAAGC
	534-584	ACTCACATGTTAAACAGGCTGCCTTGAACTCACAGAGATCTTCCTGCCTC
	907-957	CTCTCCGGATATATGAAACTGCAGAATGTGTAGCAGTGGCAAAGTTCCCA
	1113-1163	AAAGGGACCTAGAAAGGTCGCCATCTTTGACTGGGGCTGCATTTCCAAAG
	2352-2402	TGGAAGTGGATATAAGGCAGGTGAAGATGGGAGTCCCTGTCGCCTAGTTA
	2898-2948	AATCTCGAGCCTAAAAAGTTGCACTCGGACAGCTGGCGGGATCCGAAAGA
200-kDa	811-861	CAATAAAGGATATAAAAGAGAAAGAAGACAAAATGAGGATCTCATACATT
	2529-2579	GATCTCACTTTTTAAAATTGGGGATTATAAACATATTTGTAAATAACAAA

Appendix C. Transcription start sites (TSS) prediction by Web Promoter Scan program

Isoform	Transcription factor	Location	Binding Sequence
90-92-kDa	AABS_CS2 (A-activatorbinding site)	2560	GTGNNGYAA
	Sp 1 (Specificity Protein 1)	2728	GGGGGCGG
	T-Ag (T antigen)	2729	GGGGC
	Sp1	2730	GGGCGG
140-kDa	None		
200-kDa	None		
