# Supplementary material for: Cloning and promoter analysis of palladin 90-kDa, 140-kDa, and 200-kDa isoforms involved in skeletal muscle cell maturation
Source: BMC Res Notes. 2020 Jul 3;13:321. doi: 10.1186/s13104-020-05152-9 (PMC7333403; doi:10.1186/s13104-020-05152-9)
Supplement: Supplementary file 3 — Additional file 3: Figure S1. Transcription factors general expression. [file 13104_2020_5152_MOESM3_ESM.pdf]

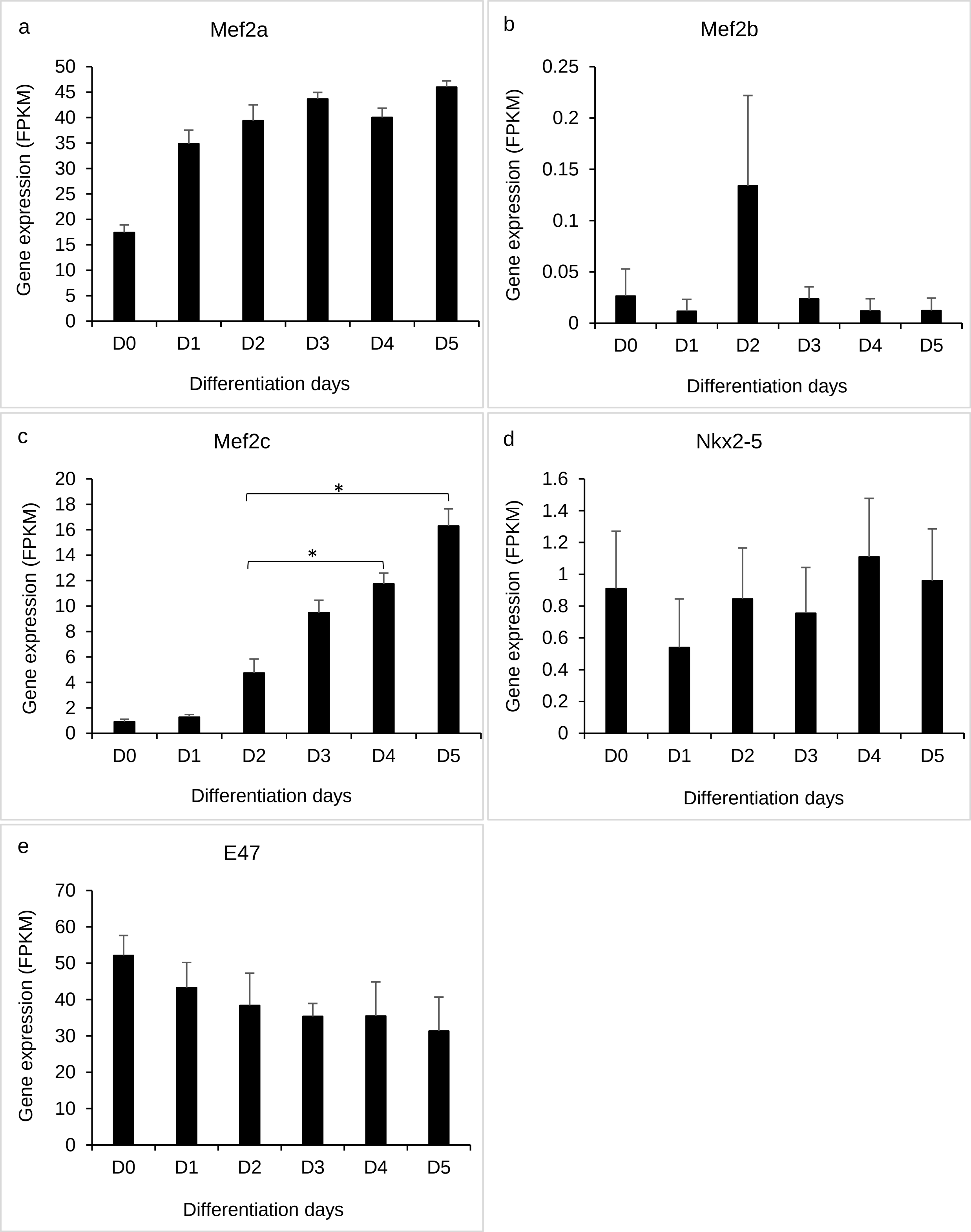
a
Mef2a
Gene expression (FPKM)
0
5
10
15
20
25
30
35
40
45
50
D0
D1
D2
D3
D4
D5
Differentiation days
b
Mef2b
Gene expression (FPKM)
0
0.05
0.1
0.15
0.2
0.25
D0
D1
D2
D3
D4
D5
Differentiation days
c
Mef2c
Gene expression (FPKM)
0
2
4
6
8
10
12
14
16
18
20
*
*
D0
D1
D2
D3
D4
D5
Differentiation days
d
Nkx2-5
Gene expression (FPKM)
0
0.2
0.4
0.6
0.8
1
1.2
1.4
1.6
D0
D1
D2
D3
D4
D5
Differentiation days
e
E47
Gene expression (FPKM)
0
10
20
30
40
50
60
70
D0
D1
D2
D3
D4
D5
Differentiation days

Additional file 3.

Figure S1. Transcription factors general expression. Expression of specific transcription factors (a) Mef2a, (b) Mef2b, (c) Mef2c, (d) Nkx2-5, and (e) Tcf3 (E47) which binding site have been identified on the palladin promoter region. Data was presented as Fragments Per Kilobase of transcript per Million mapped reads (FPKM) ±SEM, n=3 independent biological replicates. (*) p value < 0.05
