# Supplementary material for: Cloning and promoter analysis of palladin 90-kDa, 140-kDa, and 200-kDa isoforms involved in skeletal muscle cell maturation
Source: BMC Res Notes. 2020 Jul 3;13:321. doi: 10.1186/s13104-020-05152-9 (PMC7333403; doi:10.1186/s13104-020-05152-9)
Supplement: Supplementary file 4 — Additional file 4: Figure S2. Expression of palladin isoforms and myogenic genes. [file 13104_2020_5152_MOESM4_ESM.pdf]

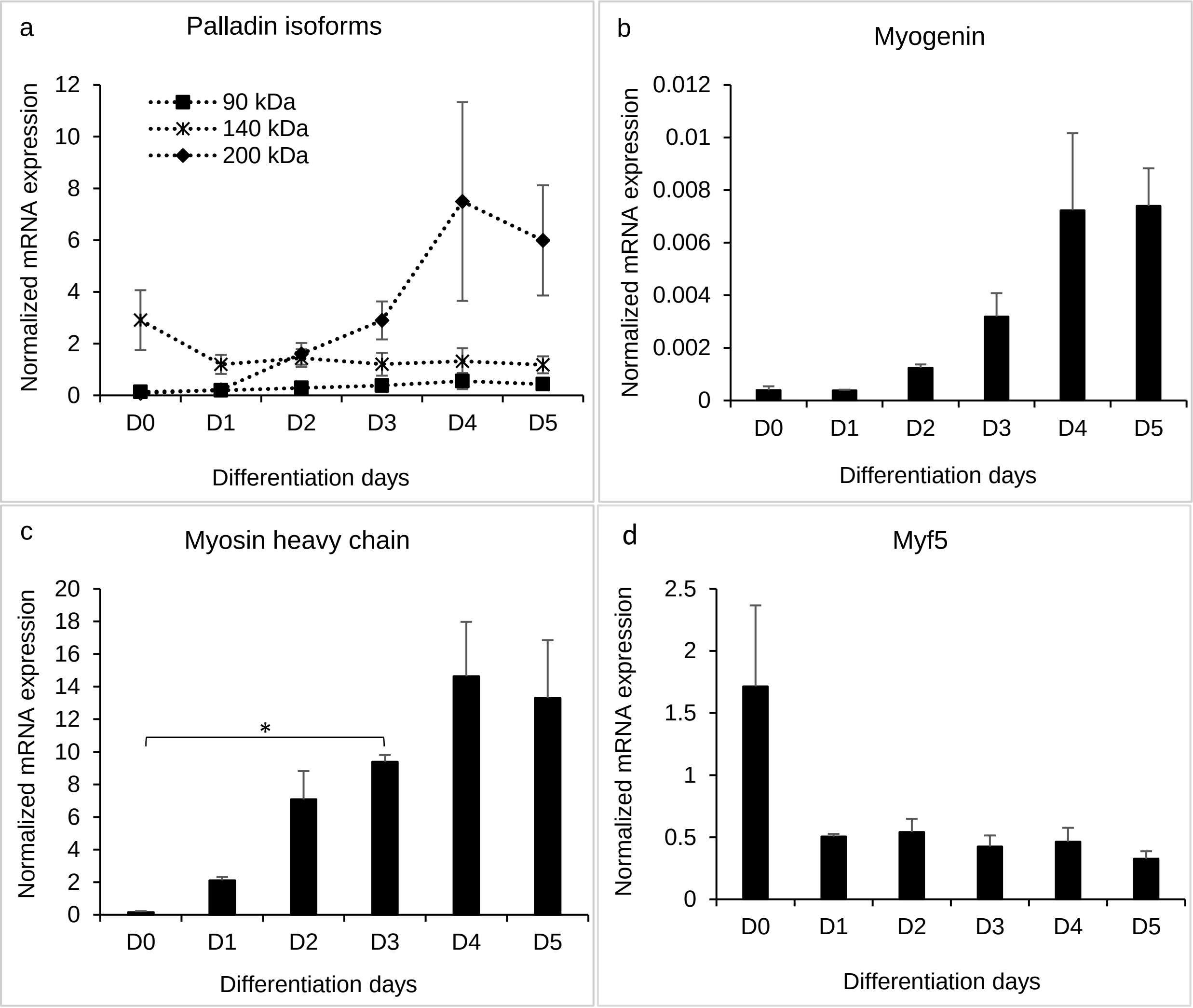

Additional file 4.

Figure S2. Expression of palladin isoforms and myogenic genes. The expression profiles of (a) Palladin isoforms, (b) Myogenin, (c) Myosin heavy chain, and (d) Myf5 were analyzed by qPCR using total RNA extracted from C2C12 cells on separated differentiation days. The values were normalized against the Ap3d1 internal control and presented as mean ±SEM of three independent biological replicates (n=3). (*) *p* value < 0.05
